# Sonographic findings of acute hepatitis in the emergency department

**DOI:** 10.1002/ccr3.6046

**Published:** 2022-08-24

**Authors:** Jessica Koehler, Lori Stolz, Patrick Minges

**Affiliations:** ^1^ Department of Emergency Medicine Univeristy of Michigan Ann Arbor Michigan USA; ^2^ Department of Emergency Medicine University of Cincinnati College of Medicine Cincinnati Ohio USA

**Keywords:** gallbladder wall, ultrasound, viral hepatitis

## Abstract

Viral hepatitis is a common cause of upper abdominal pain, vomiting, and abnormal liver function tests. In 2018, there was an outbreak of hepatitis A cases in southwestern Ohio, one of many across the United States in recent years. Viral hepatitis can demonstrate impressive gallbladder wall edema and thickening on ultrasound imaging. We describe a case series where marked gallbladder wall thickening was noted on point‐of‐care ultrasound and either led to the correct diagnosis or prompted some diagnostic uncertainty. In an undifferentiated patient, this may confuse the clinical picture because most emergency physicians may not be aware of this finding.

## Abstract 2

There has long been a description of alternative etiologies of gallbladder wall thickening and edema within the radiology body of literature including acute hepatitis; this knowledge has not been disseminated in the emergency medicine literature.

## 1 INTRODUCTION

Acute viral hepatitis is a highly contagious viral infection that may present with subclinical symptoms or with anorexia, nausea, abdominal pain, jaundice, and dark urine.^1^, ^2^

In 2017, large person‐to‐person outbreaks began occurring in primarily in patients using intravenous drugs and the homeless population, populations that are likely to seek care in the emergency department (ED).^3^ Right upper quadrant (RUQ) pain is a common presentation and the use of point‐of‐care ultrasound (POCUS) to evaluate for cholelithiasis and cholecystitis is common in the ED. An understanding of the sonographic findings of acute hepatitis is important for all practitioners of RUQ POCUS as the gallbladder wall thickening seen may mimic acute cholecystitis and lead to inappropriate treatment or additional work‐up.

## 2 CASE SERIES

### 2.1 Case 1

**FIGURE 1:**
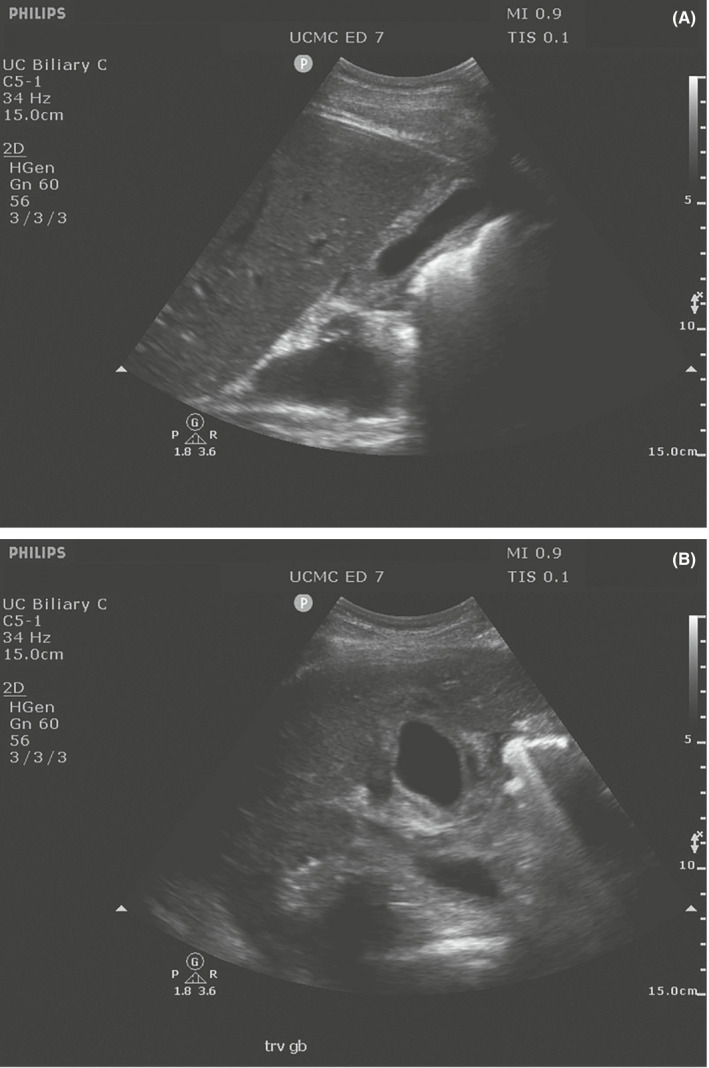
(A) Case 1. Point‐of‐care ultrasound of gallbladder in a longitudinal plane demonstrating a thickened gallbladder wall without any evidence of stones. (B) Case 1. A point‐of‐care ultrasound of the gallbladder in transverse plane demonstrating the double wall sign that can be seen in acute hepatitis. The double wall is a relatively less echogenic area that separates the more echogenic layers of the gallbladder wall

A 38‐year‐old male with a history of chronic hepatitis C and polysubstance abuse presented to the ED with 4 days of worsening epigastric abdominal pain associated with decreased appetite, nausea without emesis, and dark urine. At presentation, his heart rate was 78 and blood pressure was 132/66. On examination, he had scleral icterus with jaundice to his chest and abdominal tenderness to palpation. Laboratory analyses demonstrated bilirubinemia with a total of 11.4 mg/dl, direct of 7.8 mg/dl, and indirect of 3.6 mg/dl. His transaminases were elevated with an AST of 3468 U/L and an ALT of 3998 U/L. A RUQ POCUS was done by the provider to evaluate for cholelithiasis and cholecystitis, which showed gallbladder wall thickening without stones and with a double wall sign (Figure 1A,B). A CT was ordered with a viral hepatitis panel. CT demonstrated impressive thickening of the gallbladder wall. The patient's hepatitis viral panel showed new reactive IgG and IgM antibodies to Hepatitis A. He was admitted to the gastroenterology service who obtained a radiology‐performed ultrasound that described the gallbladder as “edematous with wall thickening and striated appearance.” While inpatient, the patient was managed conservatively before being discharged.

### 2.2 Case 2

A 24‐year‐old male with no significant past medical history presented to the ED with 3 days of orange urine and upper abdominal pain. The presenting vital signs showed a heart rate of 78 with a blood pressure of 130/77 and physical exam demonstrated tenderness in the RUQ. Urinalysis showed moderate bilirubin. His providers were initially concerned about kidney stones and ordered a CT with no contrast that showed a thickened gallbladder wall. These providers note in their interpretation the thickened wall but state that “the history and physical examination are not consistent with acute cholecystitis.” RUQ POCUS was performed to evaluate for the presence of cholelithiasis, and this study demonstrated a thickened gallbladder wall without surrounding pericholecystic fluid and no evidence of cholelithiasis. LFTs showed a total bilirubin of 7.2 mg/dl, with a direct of 4.8 mg/dl and indirect of 2.4 mg/dl. His transaminases were elevated, an AST of 1071 U/L and ALT of 2459 U/L. A hepatitis panel was ordered that demonstrated reactive IgM for Hepatitis A. The patient was admitted to the gastroenterology service and he was managed symptomatically prior to discharge 24 h later.

## 3 DISCUSSION

Abdominal pain is the most common chief complaint in the ED, accounting for 8.6% of all visits.^4^ The RUQ POCUS can be an integral part in the work‐up of these patients as well, especially when laboratory abnormalities are found. The two presented cases demonstrate marked gallbladder wall thickening in the setting of an acute viral hepatitis that was diagnosed in the ED. Three distinct findings have been described in regards to sonographic findings during an acute viral hepatitis infection: gallbladder wall thickening, double‐wall appearance, and sludge.^5^ The double wall is a relatively less echogenic area that separates the more echogenic layers of the gallbladder wall.

Emergency physicians may not always be acutely aware of the correlation between wall thickness and acute hepatitis. While there are many different etiologies of gallbladder wall thickening (pancreatitis, severe pyelonephritis, heart or renal failure, portal venous hypertension, and sepsis), acute hepatitis is one that has clinical overlap with cholecystitis, which can prompt physicians to falsely anchor on the wrong diagnosis.^6^ Any physician performing RUQ POCUS should be aware of this confounder in the work‐up of patients presenting with RUQ abdominal pain as it may help them better arrive at the correct diagnosis. The utility of RUQ POCUS in the ED is often centered around the diagnosis of acute cholecystitis. Failure to recognize and be aware of alternative etiologies of gallbladder wall thickening may lead to delayed diagnosis and unnecessary procedures and testing.

In conclusion, while there has long been a description of alternative etiologies of gallbladder wall thickening and edema within the radiology body of literature including acute hepatitis,^7^, ^8^ this knowledge has not been disseminated in the emergency medicine literature. With the increased frequency of hepatitis A outbreaks across the country, it is imperative for clinicians and POCUS users to be aware of this potentially confounding finding on RUQ POCUS.

## AUTHOR CONTRIBUTIONS

Koehler: Background research, initial draft, revisions. Stolz: revisions, concept, agreement on final draft. Minges: revisions, agreement on final draft.

## FUNDING INFORMATION

None.

## CONFLICT OF INTEREST

None.

## CONSENT

Written informed consent was obtained from the patient to publish this report in accordance with the journal's patient consent policy.

## Data Availability

Data sharing not applicable to this article as no datasets were generated or analysed during the current study.
